# Case Report: Actinomycetoma Caused by *Nocardia aobensis* from Lao PDR with Favourable Outcome after Short-Term Antibiotic Treatment

**DOI:** 10.1371/journal.pntd.0003729

**Published:** 2015-04-16

**Authors:** Inthanomchanh Vongphoumy, David A. B. Dance, Sabine Dittrich, Julie Logan, Viengmon Davong, Sayaphet Rattanavong, Joerg Blessmann

**Affiliations:** 1 Provincial Health Department, Savannakhet, Lao People’s Democratic Republic; 2 Lao-Oxford-Mahosot Hospital Wellcome Trust Research Unit, Microbiology Laboratory, Mahosot Hospital, Vientiane, Lao People’s Democratic Republic; 3 Centre for Tropical Medicine and Global Health, Nuffield Department of Medicine, University of Oxford, Oxford, England, United Kingdom; 4 Molecular Identification Services Unit, Public Health England, London, United Kingdom; 5 Bernhard Nocht Institute for Tropical Medicine, Hamburg, Germany; University of California San Diego School of Medicine, UNITED STATES

## Abstract

**Background:**

Mycetoma is a neglected, chronic, localized, progressively destructive, granulomatous infection caused either by fungi (eumycetoma) or by aerobic actinomycetes (actinomycetoma). It is characterized by a triad of painless subcutaneous mass, multiple sinuses and discharge containing grains. Mycetoma commonly affects young men aged between 20 and 40 years with low socioeconomic status, particularly farmers and herdsmen.

**Methodology / Principal Findings:**

A 30 year-old male farmer from an ethnic minority in Phin District, Savannakhet Province, Lao PDR (Laos) developed a painless swelling with multiple draining sinuses of his right foot over a period of approximately 3 years. X-ray of the right foot showed osteolysis of tarsals and metatarsals. Aerobic culture of sinus discharge yielded large numbers of *Staphylococcus aureus* and a slow growing Gram-positive rod. The organism was subsequently identified as *Nocardia aobensis* by 16S ribosomal RNA gene sequencing. The patient received antimicrobial treatment with amikacin and trimethoprim-sulfamethoxazole according to consensus treatment guidelines. Although slight improvement was noted the patient left the hospital after 14 days and did not take any more antibiotics. Over the following 22 weeks the swelling of his foot subsequently diminished together with healing of discharging sinuses.

**Conclusion:**

This is the first published case of Actinomycetoma caused by *Nocardia aobensis* and the second case of Actinomycetoma from Laos. A treatment course of only 14 days with amikacin and trimethoprim-sulfamethoxazole was apparently sufficient to cure the infection, although long-term treatment up to one year is currently recommended. Treatment trials or prospective descriptions of outcome for actinomycetoma should investigate treatment efficacy for the different members of Actinomycetales, particularly *Nocardia* spp., with short-term and long-term treatment courses.

## Author Summary

Mycetoma is a neglected tropical disease caused either by fungi or aerobic actinomycetes. It predominantly affects poor people in remote communities, where access to health care is very limited and people usually rely on traditional treatment. The incidence of mycetoma in the Lao PDR is not known, although the first case of mycetoma from the northern part of the country was published only recently. The present case report is the second case from the Lao PDR and the first published case of actinomycetoma caused by *Nocardia aobensis*. Data from the literature and the present case suggest that far shorter courses of antibiotics than those usually recommended might be sufficient to treat actinomycetoma caused by *Nocardia* spp., although these observations need to be confirmed by further studies. Shorter treatment courses would reduce drug side effects and the financial burden on patients. The latter is particularly important for low-income countries like the Lao PDR with very limited resources for health care.

## Introduction

Mycetoma is a chronic, localized, progressively destructive, granulomatous infection caused either by fungi (eumycetoma) or by aerobic actinomycetes (actinomycetoma). The disease was added to the WHO list of neglected diseases and conditions in 2013 [1]. It is most likely acquired by traumatic inoculation of the causative organism into the subcutaneous tissue, usually of the foot although any part of the body can be affected. Mycetoma is characterized by a triad of painless subcutaneous mass, multiple sinuses and discharge containing grains [2]. It commonly affects young adults, particularly males aged between 20 and 40 years. The male/female ratio is approximately 3:1. People of low socioeconomic status and manual workers such as farmers, labourers and herdsmen are most frequently affected [3,4]. Consensus treatment guidelines for eumycetoma and actinomycetoma recommend long-term treatment over 6–12 months with antifungal or antibacterial drugs [5]. However no comparative clinical trials of treatment of this condition have ever been undertaken. It is possible that the taxonomically diverse range of causative agents of eumycetoma and actinomycetoma may require more differentiated therapeutic regimens. Here we report a case of actinomycetoma caused by *Nocardia aobensis*, which was cured after short-term antibiotic treatment.

## Materials and Methods

### Ethics statement

Written informed consent was obtained from the patient for publication of this case report and any accompanying images. The board of directors of Lao-Oxford-Mahosot Hospital Wellcome Trust Research Unit, Bernhard Nocht Institute for Tropical Medicine and Savannakhet provincial hospital approved this case report for publication.

### Medical history

A 30 year-old male farmer from an ethnic minority group in Phin District, Savannakhet Province, Lao PDR, reported the development of a painless swelling of his right foot over approximately 3 years. He had no history of trauma or animal bites. Multiple draining sinuses developed over time discharging serous fluid.

### Clinical examination

**Fig 1:**
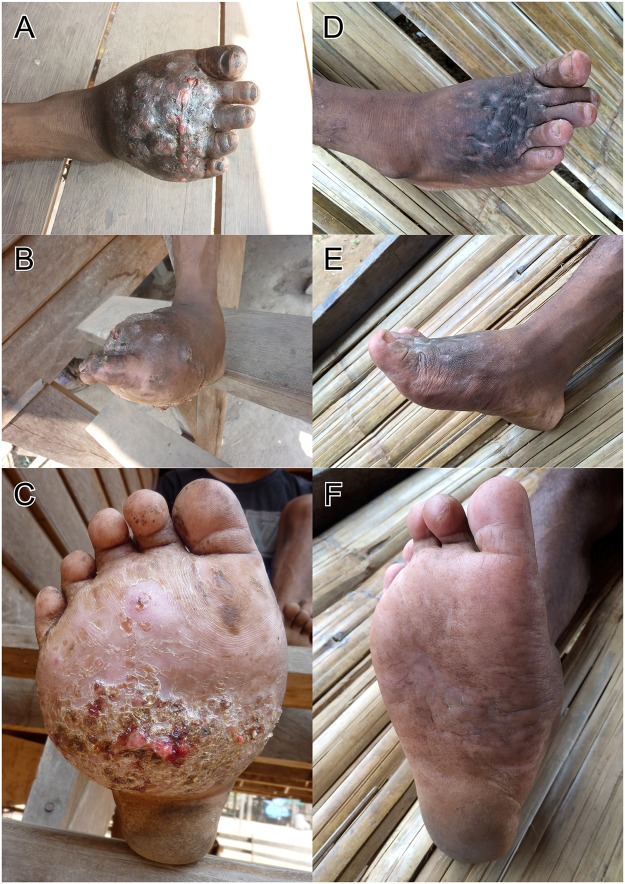
Pictures A, B and C show the right foot before treatment, Pictures D, E and F show the foot 22 weeks after treatment.

The patient was afebrile with normal vital signs and physical examination was unremarkable except for a massive tumour-like lesion of the right foot with multiple sinuses on the dorsal and plantar surfaces (Fig 1A, 1B, and 1C).

### Laboratory results before treatment

WBC: 9500/μL, Hb: 5.5 mmol/L, HCT 31%, MCV: 58 fL, PLT: 568,000/μL Lymphocytes 22%, Monocytes 14%, Granulocytes 64% Blood glucose: 4.2 mmol/l, creatinine: 53.1 μmol/l

### Laboratory results 10 months after treatment

WBC: 7000/μL, Hb: 8.9 mmol/L, HCT 45%, MCV: 71 fL, PLT: 278,000/μL Lymphocytes 24%, Monocytes 7%, Granulocytes 69% creatinine: 70.8 μmol/l

### X-ray of the right foot before treatment

**Fig 2:**
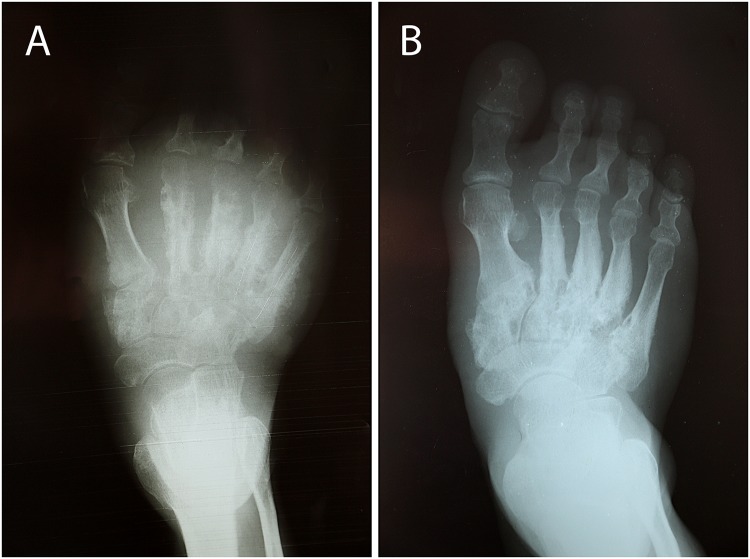
X-ray of right foot before (A) and 10 months after treatment (B).

X-ray of the right foot before treatment showed osteolysis of tarsals and metatarsal, particularly metatarsals II–V, the cuneiforms and cuboid and a pathologic fracture at the base of metatarsal V. (Fig 2A).

### X-ray of the right foot 10 months after treatment

X-ray of the right foot 10 months after treatment showed significant improvement at the metatarsal shafts II–V with healing of the fracture at metatarsal V, but still osteolysis at metatarsal bases II–V, the cuneiforms and cuboid. Joint lines between metatarsals and tarsals are not identifiable (Fig 2B).

### Microbiological and molecular biological results

**Fig 3:**
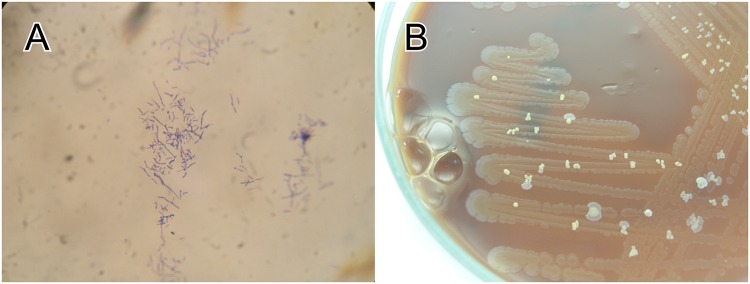
(A) Gram stain preparation showing branching Gram-positive rods, (B) Chocolate agar showing heavy growth of *Staphylococcus aureus* with scattered colonies of *Nocardia aobensis* (5 days’ incubation).

Aerobic culture of sinus discharge yielded large numbers of *Staphylococcus aureus* and a slow growing Gram-positive rod, which was apparent after 5 days’ incubation (Fig 3B). Gram stain preparation showed branching Gram-positive rods (Fig 3A). The organism was identified as *Nocardia aobensis* in two independent laboratories after amplification and sequencing of the 16S ribosomal RNA gene (839bp: coverage 839/839, identity 100%, e-value 0.00, Accession Number KP250991; 670bp [hypervariable regions V3–V7]: coverage 670/670, identity 100%, e-value 0.00, Accession Number KP404096) and blast analysis against the NCBI database [6].

### Clinical diagnosis

Mycetoma

### Microbiological diagnosis

Actinomycetoma caused by *Nocardia aobensis*

### Treatment

**Fig 4:**
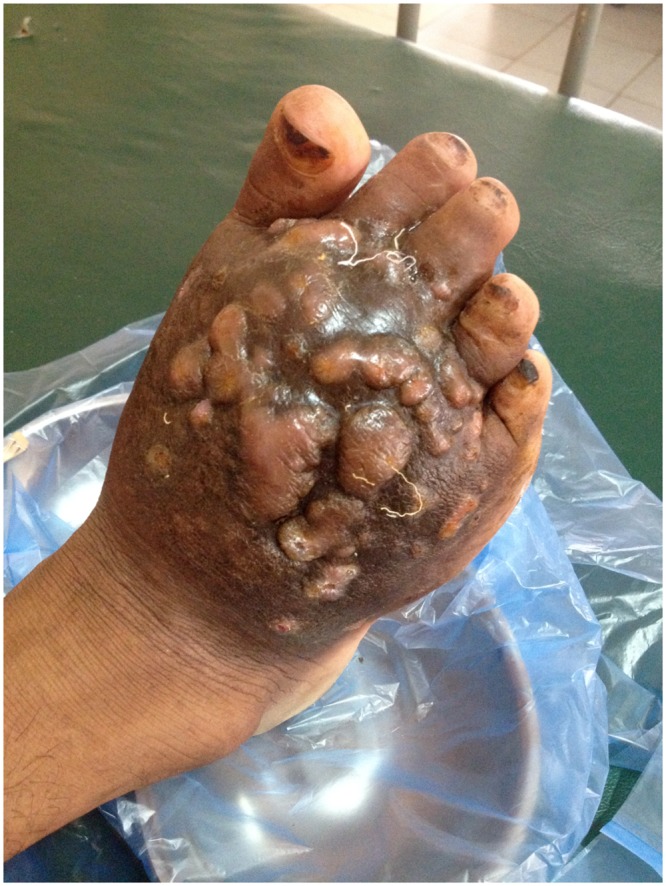
Right foot after 14 days of treatment with amikacin and trimethoprim-sulfamethoxazole. Sinuses were closed and no more discharge observed.

The patient received amikacin (15mg/kg/day) intravenously combined with trimethoprim-sulfamethoxazole (TMP-SMX) (7/35 mg/kg/day) per os for 14 days. Although slight improvement was noted at that stage, with a decrease of discharge and closure of sinuses, the patient left the hospital against medical advice for traditional treatment in his village and did not take any more antibiotics (Fig 4). Over the following weeks the swelling of his foot subsequently diminished together with healing of discharging sinuses. This was confirmed when he was seen 22 and 43 weeks after antibiotic treatment at the health centre in his village and the provincial hospital (Fig 1D, 1E, and 1F). He resumed physical work in the forest without limitation and reported a significant overall improvement of his state of health. X-ray of his right foot showed significant improvement at the metatarsal shafts, but still signs of osteolysis at the metatarsal bases, the cuneiforms and cuboid. The initial anaemia and thrombocythaemia, most likely the result of chronic infection, returned to normal 10 months after treatment.

## Discussion

The incidence of mycetoma in Laos is not known. Only one case of actinomycetoma, caused by *Actinomadura madurae*, from Xieng Khouang province in the northern part of the country has been published [7].

The patient’s history and the clinical appearance of his foot were suggestive of mycetoma and microbiological culture and molecular biological identification of *Nocardia aobensis* confirmed the diagnosis of actinomycetoma. Correct identification of the causative agent is crucial in order to choose the appropriate treatment.

More than 30 species have so far been identified as aetiological agents of mycetoma worldwide [8]. *Actinomadura madurae*, *Actinomadura pelletieri*, *Nocardia brasiliensis*, *Nocardia asteroides* and *Streptomyces somaliensis* are most frequently responsible for causing actinomycetoma [4]. The patient described is to our knowledge the first confirmed case of actinomycetoma caused by *Nocardia aobensis* and adds one more causative agent to the list. *Nocardia aobensis* was first described in 2004 after having been isolated from sputum of Japanese patients [9]. Antimicrobial susceptibility testing has not been done for the isolate described here, but most *Nocardia* spp. including *Nocardia aobensis*, are susceptible to amikacin and TMP-SMX [10].

In 1987, Welsh et al. reported a study which included 15 patients treated for actinomycetoma, all but one of which were caused by *Nocardia brasiliensis*, with cycles of amikacin alone (2 cases) and amikacin with TMP-SMX (13 cases). Treatment with amikacin 15mg/kg/day for 3 weeks together with TMP-SMX 7/35mg/kg/day for 5 weeks was defined as 1 cycle. The patients were all cured after 1–3 cycles, with 4 patients cured after 1 cycle, 8 patients cured after 2 cycles and 3 patients cured after 3 cycles [11,12]. In 1982 a young man with severe actinomycetoma and pulmonary involvement caused by *Nocardia brasiliensis* was successfully treated with one cycle [13]. Based on their experience with 56 patients Welsh et al. recommend 1–4 cycles with amikacin and TMP-SMX in severe cases of actinomycetoma that do not respond to TMP-SMX alone, achieving a cure rate of approximately 90% [5]. The number of cycles is dependent on the clinical and bacteriological response and varies from case to case. In our patient, a clinical improvement with closure of sinuses was noted after only two weeks of amikacin and TMP-SMX (Fig 4). Swelling was still significant at that time and took 22 weeks to resolve, which under normal circumstances might have misled the clinician in charge to continue the treatment for a longer period, although it appears that cure had already been achieved after 2 weeks. Current practice may thus entail some “overkill”. However, although haemoglobin and platelet count returned to normal values 43 weeks after treatment, radiological changes of metatarsal and tarsal bones had not completely disappeared at that time and this requires further follow up, because recurrence 2 years after remission has been previously reported [5].

Clinical trials or prospective descriptions with short-term and long-term antimicrobial treatment courses and longer periods of follow up are needed to inform optimal antibiotics and particularly treatment duration. Actinomycetoma affects mostly poor villagers in remote communities and shorter treatment courses would significantly reduce the financial burden and side effects of amikacin and TMP-SMX.

## Conclusion

To our knowledge this is the first published patient with actinomycetoma caused by *Nocardia aobensis* and the second case of actinomycetoma from Laos. A treatment course of only 14 days with amikacin and TMP-SMX appears to have been sufficient to cure the infection as defined by 43 weeks follow up. Trials or prospective descriptions of efficacy of different antimicrobial therapy with short-term and long-term treatment courses for actinomycetoma should investigate treatment outcome separately for different members of Actinomycetales, particularly *Nocardia* spp.

## Supporting Information

S1 Fig Gene Bank accession number 1 for nucleotide sequence. (DOCX)

S2 Fig Gene Bank accession number 2 for nucleotide sequence. (DOCX)
