# Supplementary material for: Case Report: Actinomycetoma Caused by Nocardia aobensis from Lao PDR with Favourable Outcome after Short-Term Antibiotic Treatment
Source: PLoS Negl Trop Dis. 2015 Apr 16;9(4):e0003729. doi: 10.1371/journal.pntd.0003729 (PMC4400045; doi:10.1371/journal.pntd.0003729)
Supplement: S1 Fig — (DOCX) [file pntd.0003729.s001.docx]

Dear GenBank Submitter:

Thank you for your direct submission of sequence data to GenBank.  We

have provided a GenBank accession number for your nucleotide sequence:

BankIt1788268 seq2 KP404096

The GenBank accession number should appear in any publication that reports

or discusses these data, as it gives the community a unique label with which

they may retrieve your data from our on-line servers. You may prepare and

submit your manuscript before your accession is released in GenBank.

Submissions are not automatically deposited into GenBank after being

accessioned. Each sequence record is individually examined and processed

by the GenBank annotation staff to ensure that it is free of errors or

problems.

You have not requested a specific release date for your sequence data.

Therefore, your record(s) will be released to the public database once

they are processed.  If this is not what you intended, please contact

us as soon as possible with the correct release date.

Since the flatfile record is a display format only and is not an editable

format of the data, do not make changes directly to a flatfile.  For

complete information about different methods to update a sequence record,

see:   <http://www.ncbi.nlm.nih.gov/Genbank/update.html>

Any inquiries about your submission should be sent to [gb-admin@ncbi.nlm.nih.gov](mailto:gb-admin@ncbi.nlm.nih.gov)

For more information about the submission process or the available

submission tools, please contact GenBank User Support at

[info@ncbi.nlm.nih.gov](mailto:info@ncbi.nlm.nih.gov).

Please reply using the original subject line.

This will allow for faster processing of your correspondence.

Sincerely,

Rich McVeigh Ph.D.

Contractor

The GenBank Direct Submission Staff

Bethesda, Maryland USA

*******************************************************************

[gb-admin@ncbi.nlm.nih.gov](mailto:gb-admin@ncbi.nlm.nih.gov) (for updates/replies to GenBank entries)

[info@ncbi.nlm.nih.gov](mailto:info@ncbi.nlm.nih.gov)     (for general questions regarding GenBank)

www.ncbi.nlm.nih.gov/books/NBK51157/  GenBank Submissions Handbook

******************************************************************
